# Hydrolysis and Photolysis Kinetics, and Identification of Degradation Products of the Novel Bactericide 2-(4-Fluorobenzyl)-5-(Methylsulfonyl)-1,3,4-Oxadiazole in Water

**DOI:** 10.3390/ijerph15122741

**Published:** 2018-12-05

**Authors:** Xingang Meng, Lingzhu Chen, Yuping Zhang, Deyu Hu, Baoan Song

**Affiliations:** State Key Laboratory Breeding Base of Green Pesticide and Agricultural Bioengineering, Key Laboratory of Green Pesticide and Agricultural Bioengineering, Ministry of Education, Guizhou University, Guiyang 550025, China; gs.xgmeng14@gzu.edu.cn (X.M.); lzchen@gzu.edu.cn (L.C.); ypzhang2@gzu.edu.cn (Y.Z.)

**Keywords:** Fubianezuofeng, bactericide, kinetics, mechanism, water, abiotic degradation

## Abstract

Hydrolysis and photolysis kinetics of Fubianezuofeng (FBEZF) in water were investigated in detail. The hydrolysis half-lives of FBEZF depending on pH, initial concentration, and temperature were (14.44 d at pH = 5; 1.60 d at pH = 7), (36.48 h at 1.0 mg L^−1^; 38.51 h at 5.0 mg L^−1^; and 31.51 h at 10.0 mg L^−1^), and (77.02 h at 15 °C; 38.51 h at 25 °C; 19.80 h at 35 °C; and 3.00 h at 45 °C), respectively. The photolysis half-life of FBEZF in different initial concentrations were 8.77 h at 1.0 mg L^−1^, 8.35 h at 5.0 mg L^−1^, and 8.66 h at 10.0 mg L^−1^, respectively. Results indicated that the degradation of FBEZF followed first-order kinetics, as the initial concentration of FBEZF only had a slight effect on the UV irradiation effects, and the increase in pH and temperature can substantially accelerate the degradation. The hydrolysis Ea of FBEZF was 49.90 kJ mol^−1^, which indicates that FBEZF belongs to medium hydrolysis. In addition, the degradation products were identified using ultra-high-performance liquid chromatography coupled with an Orbitrap high-resolution mass spectrometer. One degradation product was extracted and further analyzed by ^1^H-NMR, ^13^C-NMR, ^19^F-NMR, and MS. The degradation product was identified as 2-(4-fluorobenazyl)-5-methoxy-1,3,4-oxadiazole, therefore a degradation mechanism of FBEZF in water was proposed. The research on FBEZF can be helpful for its safety assessment and increase the understanding of FBEZF in water environments.

## 1. Introduction

**Figure 1:**
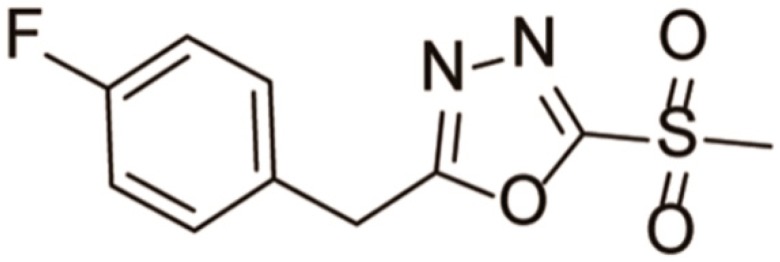
Chemical structure of FBEZF.

Rice bacterial leaf blight caused by pathogen *Xanthomonas oryzae pv. oryzae* (Xoo) is the most important bacterial disease of rice in the rice-growing period. Fubianezuofeng (FBEZF, 2-(4-fluorobenzyl)-5-(methylsulfonyl)-1,3,4-oxadiazole, Figure 1) is a novel bactericide that exhibits considerable inhibition effects against rice bacterial leaf blight and leaf streak with half-maximal effective concentration (EC_50_) values of 1.07 µg/mL and 7.14 µg/mL, respectively, which are superior to commercial agents such as bismerthiazol and thiadiazole copper [1]. FBEZF has been developed as a new oxadiazole sulfone bactericide and is classified as a sulfone derivative. Field trials have been performed in 2015, and the results show that FBEZF has potent control efficiency against rice bacterial leaf blight in China. Owing to the potential development prospect of FBEZF in China, rapid and sensitive methods for detection of byproducts and the degradation studies on FBEZF are required.

Hydrolysis and photolysis are crucial in the environmental behavior of pesticides. In the last decades, many studies have reported the photolysis of pesticides [2,3,4,5,6] and effect of organic and inorganic compounds in the environment on the degradation of pesticides [7,8]. The photocatalytic degradation of 16 substituted phenylurea pesticides in water has been studied, 13 degradation products have been identified, and a degradation mechanism proposed that indicates dealkylation is the main degradation pathway [9]. Photolysis of bromoxynil and trifluralin has been reported by numerous researchers [10,11,12,13,14]. For example, Chelme-Ayala et al. [15] found that hydroxylation and debromination were the primary pathways for bromoxynil degradation, whereas hydroxylation and dealkylation were the major degradation mechanisms of trifluralin. Moreover, the hydrolysis of pesticides was reported in some papers. Wyer et al. [16] proposed the enhanced hydrolysis of diazinon, in which bidentate binding of Ag^+^ to S of the P = S electrophilic site in tandem with binding to N of the leaving group stabilizes the SN_2_ (P) transition state relative to the ground state. Zhang et al. [17] investigated the hydrolysis of chlorpyrifos and diazinon in aqueous solution under ultrasonic irradiation. The hydrolysis, oxidation, hydroxylation, dehydration, and decarboxylation were deduced to contribute to the degradation reaction and the degradation pathway for both pesticides.

Temperature and pH are the factors that can influence the degradation of pesticides [18,19]. Soil degradation of the fungicide chlorothalonil (2,4,5,6-tetrachloroisophthalonitrile or TPN), was studied under laboratory conditions. Although the dissipation was less at 30 °C and larger at 38 °C than that at 25 °C, the dissipation rate of TPN increased with temperature [20]. The effect of temperature on the degradation of 1-benzyltriazole and 4-fluoro fungicides was reported. The degradation rate of the unsubstituted compound was sensitive to temperature changes, increasing eightfold as the temperature rose from 5 °C (T_1/2_ = 240 days) to 10 °C (T_1/2_ = 34 days) [19]. Kinetic studies on the degradation of aldrin, endosulfan, and lindane were reported under various temperatures and pH, and the changes in pH and temperature influenced their degradation [21]. The degradation of methyl parathion showed that the degradation rate increased as the pH level increased from 3.0 to 9.0 [22]. Isobutylurea was neither photolytic nor hydrolytic in water [23]. Xu et al. [24] reported that the dissipation rates of isobutylurea were not affected by the increase in pH value from 6 to 11. The degradation rate of boscalid was increased with pH, rapidly proceeding in alkaline aqueous solution [25].

To the best of our knowledge, no research on the degradation products and mechanism of FBEZF in aqueous solution has been published. Only one proteomic analysis of FBEZF in *Xanthomonas axonopodis pv. citri* was found [26]. In terms of analysis, only one article reported residue pretreatment and JHXJZ residue (similar structure to FBEZF) in tomato [27]. In addition, after FBEZF is applied to the field, may find its way into drinking water through runoff, leaching, or osmosis. Water is essential to the survival of our human. FBEZF whether degradation in water, degradation of security or not is the problem to be solved. So describing degradation kinetics, potential degradation products, and the degradation mechanism of FBEZF in water is necessary. The objectives of the present study are as follows: (1) to demonstrate the degradation kinetics of FBEZF in water under different conditions, (2) to investigate the photolysis of FBEZF in water, and (3) to elucidate the potential degradation intermediates and mechanism of FBEZF degradation in water.

## 2. Materials and Methods

### 2.1. Chemicals and Reagents

An analytical standard of FBEZF (99.0% purity) was provided by the Key Laboratory of Green Pesticide and Agricultural Bioengineering, Ministry of Education, Guizhou University (Guiyang, China). HPLC-grade acetonitrile and methanol were purchased from Merck (Darmstadt, Germany). Analytical-grade methylene chloride, ethyl acetate, petroleum ether, methanol, potassium biphthalate (KHP), KH_2_PO_4_, Na_2_B_4_O_7_·10H_2_O, KCl and NaOH were purchased from Jinshan Chemical Reagent Co. (Chengdu, China). Distilled water was obtained from Watsons Co. Ltd. (Dongguan, China). Syringe filters (nylon, 0.22 µm) were purchased from PeakSharp Technologies (Yibin, China).

### 2.2. UPLC Analysis

The detection of FBEZF in water was performed on a Waters ACQUITY UPLC H-class system fitted with a sample manager, a quaternary solvent manager, a PDA detector, and an ACQUITY UPLC BEH Shield RP18 column (50.0 mm × 2.1 mm i. d., 1.7 μm film thickness) (Waters Corporation, Milford, MA, USA). The column temperature was at 40 °C. A total of 2 µL sample solution was injected, and the chromatography was run with acetonitrile/water (30/70, *v*/*v*) at a flow rate of 0.2 mL min^−1^. The chromatographic conditions were determined from the trial experiments for optimal results in terms of peak shape, column efficiency, chromatographic analysis time, selectivity, and resolution. FBEZF was detected at 212 nm. Retention time of FBEZF was 3.9 min under the optimized chromatographic conditions.

### 2.3. UPLC–MS/MS Analysis

FBEZF and its degradation products in water were separated on an UltiMate 3000 ultra-high-performance liquid chromatography (UPLC) system (Thermo Scientific Transcend, Thermo Fisher Scientific, San Jose, CA, USA) coupled with a single-stage Orbitrap high-resolution mass spectrometer (MS/MS) (Q-Exactive, Thermo Fisher Scientific, Bremen, Germany). The experiment sample was detected with a heated electrospray interface (ESI, Thermo Fisher Scientific,) in positive ion mode (ESI^+^). Xcalibur program version 3.0.63 (Thermo Fisher Scientific) with Qual and Quanbrowser was used to process the data. Thermo Scientific Dionex Chromeleon 6.8 was employed to screen the target compounds. Optimized tuning parameters were as follows: aux gas heater temperature at 300 °C; capillary temperature at 300 °C; spray voltage at 3.70 kV; and sheath, auxiliary, and sweep gas flow rates at 35, 10, and 2 a.u., respectively. UPLC separations were obtained using an ACQUITY UPLC BEH Shield RP18 column (50.0 mm × 2.1 mm i.d., 1.7 μm film thickness). The mobile phase comprised component A accounting for 70% (H_2_O + 0.1% formic acid) and component B was 30% (CH_3_CN). The injection volume was 5 µL and the flow rate was set at 0.2 mL min^−1^. Data were collected in a positive mode within the range of 150 *m*/*z* to 500 *m*/*z* using full scan and t-SIM/ddMS^2^ analysis with resolution 140,000 during the entire process.

### 2.4. Calibration Curve

For the quantification experiment of FBEZF, a calibration curve was established by analyzing the peak areas of FBEZF at concentrations of 0.105, 0.510, 1.05, 5.10, 10.5, 20.2, and 45.0 mg L^−1^. The curve has a good linear correlation coefficient (>0.9999) with a regression equation of *y* = 27789*x* − 3813.7 (*y* = peak area; *x* = concentration, mg L^−1^). The LOD and LOQ of FBEZF in water were 0.0015 mg L^−1^ and 0.005 mg L^−1^, respectively.

### 2.5. Degradation Kinetics Experiments

Photolysis experiments of FBEZF were conducted in a climate chamber with a 30 W UV lamp. The photon fluxes of 30 W UV lamps were 41.03 μmol m^−2^ s^−1^. Then, these photolysis experiments were performed in 250 mL quartz flasks with different initial concentrations of FBEZF (1.0, 5.0, and 10.0 mg L^−1^) aqueous solution. Hydrolysis experiments of FBEZF were conducted in 500 mL wide-mouth bottles in the dark. The effect of pH, temperature, and different initial concentrations on the hydrolysis of FBEZF was investigated. All laboratory glassware was sterilized and 0.1 g NaN_3_ was added into the aqueous solution to prevent the growth of bacteria. The samples were filtered with 0.22 µm syringe filters for UPLC analysis. All experiment results were calculated on the average of triplicate experiments.

### 2.6. Identification of Degradation Products

Standard FBEZF was directly dissolved in the water until a concentration of 100 mg L^−1^ was reached to obtain the detectable signals of potential degradation products on the UPLC–MS/MS system. When most FBEZF have been degraded, the samples were filtered with 0.22 µm syringe filters and then detected on the UPLC–MS/MS system. These potential products were further analyzed to elucidate their structures using the UPLC–MS/MS via retention times, MS, MS^2^, and observed mass differences compared with those of FBEZF.

The degradation products of FBEZF were isolated and analyzed in this experiment. FBEZF (1 g) was added to the water to obtain a sufficient amount of the degradation product. Liquid–liquid extraction was selected for the extraction of the degradation products. The hydrolyzed sample was extracted multiple times by dichloromethane. Extraction of the sample was concentrated and purified by thin-layer chromatography (TLC) (ethyl acetate/petroleum ether, 3/1, *v*/*v*). The products were further analyzed by ^1^H-NMR, ^13^C-NMR, ^19^F-NMR, and MS.

## 3. Results and Discussion

### 3.1. Hydrolysis Experiments

#### 3.1.1. Effect of pH

**Figure 2:**
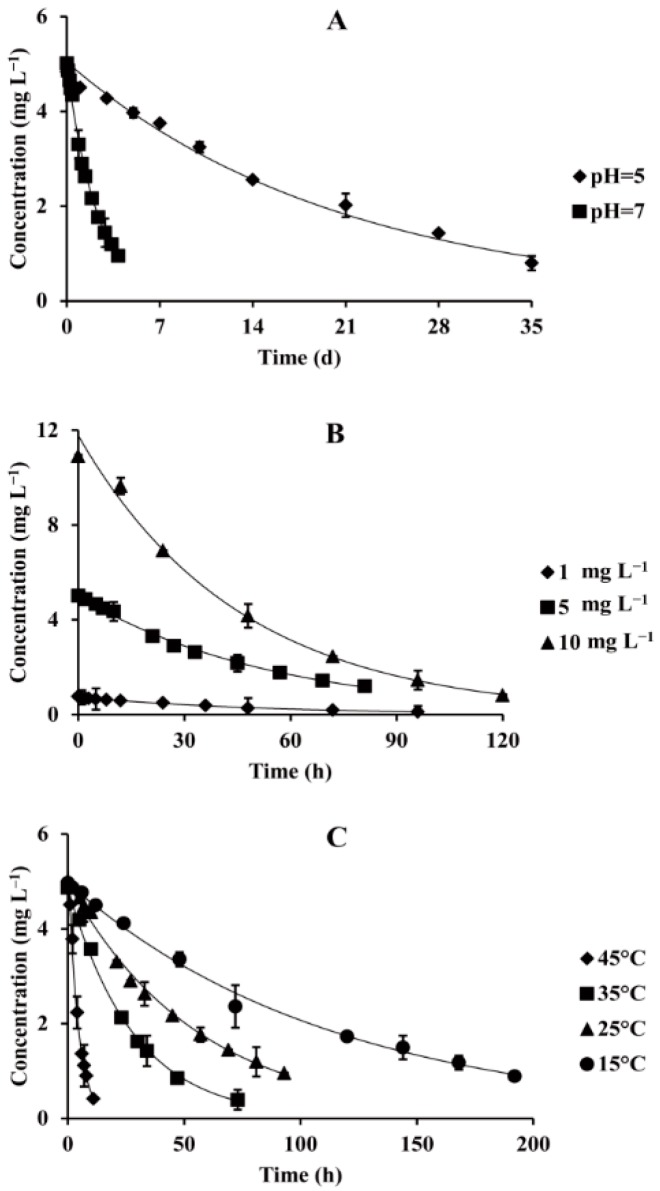
Effect of different pH values ((**A**), spiked at 5 mg L^−1^, 25 °C), initial concentrations ((**B**), 25 °C, pH = 7), and temperatures ((**C**), spiked at 5 mg L^−1^, pH = 7) on the hydrolysis of FBEZF in water.

The experimental results of the degradation kinetics are listed in Figure 2A and Table S1. The half-lives of FBEZF in buffer solutions were 14.44 d at pH = 5 and 1.60 d at pH = 7. The results indicated that FBEZF was relatively stable in acidic solution but unstable in alkaline solution, allowing the hydrolysis ratio to reach nearly 100% in pH 9 buffer solution after 25 min. Therefore, pH values played a critical role in the degradation rate of FBEZF in water because the degradation rate decreases with pH, and FBEZF rapidly degrades in alkaline aqueous solutions. Because the degradation rate of FBEZF in water environment is relatively fast, it won’t cause harm for the environment and human health.

#### 3.1.2. Effect of Initial Concentration

The effect of different initial concentrations on the hydrolysis rate of FBEZF and the corresponding kinetic parameters are shown in Table S2. Figure 2B shows that the half-lives of FBEZF in different initial concentrations were 36.48 h (1.0 mg L^−1^), 38.51 h (5.0 mg L^−1^), and 31.51 h (10.0 mg L^−1^). The experimental results also demonstrate that the initial concentration of FBEZF only had a slight effect on the degradation rate. Similar results have been reported on the degradation of acephate, dufulin, and monocrotophos [28,29,30].

#### 3.1.3. Effect of Temperature

The data are listed in Table S3 and Figure 2C. The results indicate that the half-lives of FBEZF were 77.02 h at 15 °C, 38.51 h at 25 °C, 19.80 h at 35 °C, and 3.00 h at 45 °C. The hydrolysis rate increased 1.92–2.02 times with every 10 °C increase in temperature between 15 °C and 35 °C. An increase in temperature leads to a high reaction rate within a certain range [31,32]. Some researchers have indicated that the elevated temperature can result in the reduction of surface tension and threshold intensity required to produce cavitation, thus leading to the increasing degradation efficiency [33]. The effects of temperature on FBEZF degradation in aqueous solutions followed Van’t Hoff theory that the hydrolysis rate usually doubled with every 10 °C increase in temperature [34].

The temperature dependence of the rate constant k for the process is described by the Arrhenius equation as follows:

(1) $$K=A\cdot e^{-\frac{\mathrm{Ea}}{\mathrm{RT}}},\;$$

(2) $$\mathrm{lnK}=\mathrm{lnA}-\frac{\mathrm{Ea}}{\mathrm{RT}},$$

(3) ΔH=Ea−RT, 

(4) $$\Delta S=R\left(\mathrm{lnA}-\frac{K_{B}T}{h}\right),$$

where A is constant, K_B_ is Boltzmann’s constant, h is Planck’s constant, K is the rate constant obtained by the experiment, T is the absolute temperature of the experiment, and R is gas constant. The energy of activation (Ea), enthalpy of activation (ΔH), and entropy of activation (ΔS) were obtained by the preceding formula. Thermodynamic parameters of FBEZF’s hydrolysis at four temperatures are listed in Table 1. 

A plot of ln k against 1/T provided a linear line in the temperature range 288–318 K and yielded the Arrhenius expression ln k = −6103.4 (1/T) + 16.464. The Ea and ΔH were calculated as 49.90 and 47.38 kJ mol^−1^, respectively. Ea and ΔH determined the occurrence rate of pesticide hydrolysis. The large activation energy means it has a large energy difference between ground and transition states. Because few reacting molecules collided with sufficient energy to climb the high activation energy barrier resulted in a slow reaction. The low activation energy means it has a small energy difference between ground and transition states. Because reacting molecules were sufficiently energetic to climb to the activation energy barrier resulted in rapid reaction velocity. According to classification of hydrolysis [35], the hydrolysis of compound was easy at room temperature when Ea is less than 33.49 kJ mol^−1^, and the hydrolysis of compound was difficult when Ea is greater than 167.5 kJ mol^−1^. The hydrolysis Ea of FBEZF was 49.90 kJ mol^−1^, indicating the hydrolysis of FBEZF ability among the above classification. Furthermore, activation entropy (ΔS) was crucial in hydrolysis reaction because it was a measure of the degree of order. The results (Table 1) indicated that ΔS gradually decreased with the increase in temperature and reactant molecules had a greater degree of freedom than that of activation complex molecules in the hydrolysis reaction process.

### 3.2. Photolysis Experiments

#### Effect of Initial Concentration

**Figure 3:**
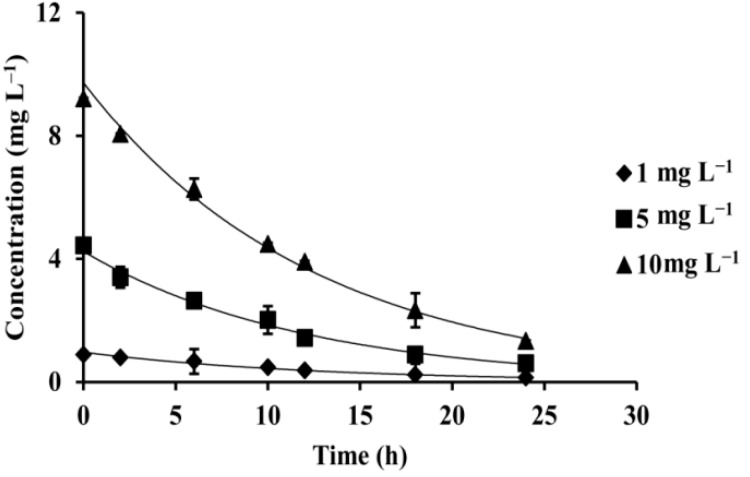
Effect of different initial concentrations on the photolysis of FBEZF in water (pH = 7).

The photolysis experimental data are listed in Figure 3 and Table S4. The photolysis experimental results showed that the half-lives of FBEZF in 1.0, 5.0, and 10.0 mg L^−1^ were 8.77, 8.35, and 8.66 h, respectively. These results were similar to the hydrolysis data of FBEZF in various initial concentrations. The photolysis experimental results also revealed that the initial concentration of FBEZF only had a slight effect on the degradation rate.

### 3.3. Identification of Degradation Product

**Figure 4:**
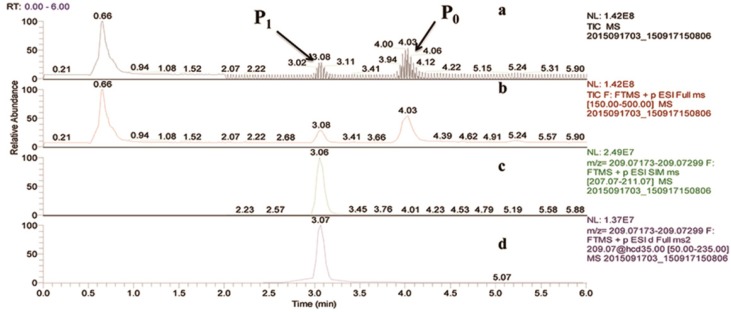
(**a**) Total ion current chromatogram of FBEZF degradation sample, (**b**) Full mass chromatogram, (**c**) Mass range chromatogram MS at *m*/*z* 209.07236, and (**d**) Mass range chromatogram MS^2^ at *m*/*z* 209.07236.

During the degradation experiments, the degradation products of FBEZF were characterized on the high-resolution MS system. Two peaks were regarded as potential degradation products by comparing the UPLC–MS/MS profiles of the degradation and the blank control samples. The peaks were identified by retention times and protonated molecular ions as follows: t = 3.08 min, *m*/*z* 209.07236, labeled P_1_; t = 4.03 min, *m*/*z* 257.03918, labeled P_0_ (Figure 4). The degradation products were identified by MS, MS^2^ of fragmentation of the protonated molecular ions or potassium-adducted ions, which were used to illuminate the structures of degradation products. For the second peak, the retention time, MS, and MS^2^ of P_0_ were the same as FBEZF, thereby confirming P_0_ as FBEZF.

**Figure 5:**
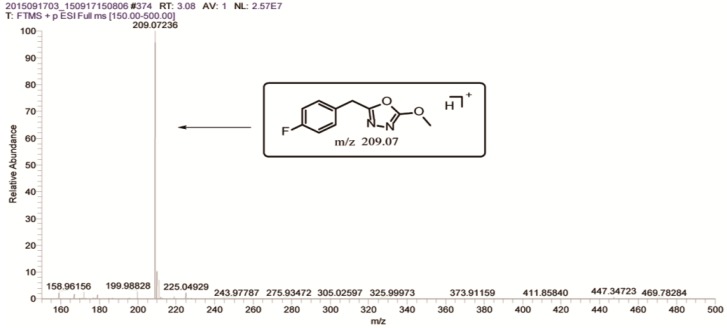
Mass spectrum of MS of P_1_ at *m*/*z* 209.0723.

**Figure 6:**
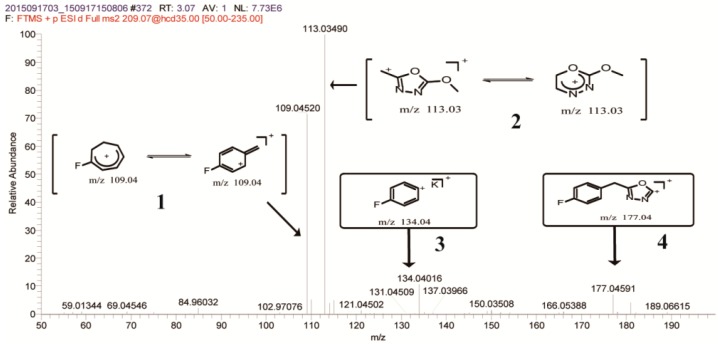
Mass spectrum of MS^2^ of P_1_ at *m*/*z* 209.07236.

P_1_ had *m*/*z* of 209.07236; if it was a protonated ion, then the molecular weight (MW) should be 208.0. According to the molecular weight, the degradation product of FBEZF has been previously assumed as 2-(4-fluorobenzyl)-5-methoxy-1,3,4-oxadiazole (Figure 5). The fragmentation pattern of P_1_’s MS^2^ was studied to confirm product P_1_. Four fragment ions at *m*/*z* 109.04520 (**1**), *m*/*z* 113.03490 (**2**), *m*/*z* 134.04016 (**3**), and *m*/*z* 177.04591 (**4**) were discovered (Figure 6).

The fragment ion at *m*/*z* 109.04520, which appeared in the MS^2^ spectrum of P_1_, was considered by a protonated molecule cleavage of an oxazole ring C–C bond. Corresponding protonated molecular fragment ion at *m*/*z* 113.03490 and a potassium-adducted fragment ion at *m*/*z* 134.04016 represented such a cleavage by one protonated molecular cleavage of a benzene ring C–C bond. The two protonated molecular fragment ions formed tropylium cation. Another ion at *m*/*z* 177.04591 was due to the loss of one −OCH_3_, indicating that P_1_ had one −OCH_3_. Therefore, P_1_ was tentatively confirmed as 2-(4-fluorobenzyl)-5-methoxy-1,3,4-oxadiazole.

Liquid–liquid extraction and TLC were selected for the extraction of degradation product to further confirm the degradation product of FBEZF. The product was then analyzed by ^1^H-NMR (Figure S1), ^13^C-NMR (Figure S2), ^19^F-NMR (Figure S3), and MS spectra (Figure S4). Figure S4 shows that the product had a molecular ion at *m*/*z* 209.0 and a sodium-adduct ion at *m*/*z* 231.0. The ^1^H-NMR, ^13^C-NMR, and ^19^F-NMR data of the degradation product were as follows: ^1^H-NMR (500 MHz, DMSO–*d*_6_, ppm) *δ*: 7.34 (dd, *J* = 8.6, 5.6 Hz, 2H, Ar–H), 7.15 (t, *J* = 8.8 Hz, 2H, Ar–H), 4.12 (s, 2H, Ar–CH_2_–), 4.06 (s, 3H, –OCH_3_); ^13^C-NMR (125 MHz, DMSO–*d*_6_, ppm) *δ*: 166.58 (s), 162.86 (s), 161.63 (s), 160.93 (s), 131.29–131.13 (m), 130.84 (s), 115.81 (s), 59.86 (s), 30.72 (s); ^19^F-NMR (471 MHz, DMSO–*d*_6_) δ 115.46.

The degradation product (P_1_) was thus confirmed as 2-(4-fluorobenzyl)-5-methoxy-1,3,4-oxadiazole. Moreover, based on the study of degradation product identification, a probable degradation mechanism was proposed. Degradation of FBEZF in water involves a nucleophilic attack on the sulfone group. Then, the intermediate combined with methanol and formed the degradation product by the loss of one H_2_O. These results can explain the relative instability of FBEZF in alkaline conditions.

### 3.4. Future Research

First, the experimental results also demonstrate that the initial concentration of FBEZF only had a slight effect on the degradation rate, but kinetic half-lives are expected to depend on first order kinetic rate coefficients rather than initial concentrations. Through the experiment proved that the degradation rate is related to the initial concentration or not is necessary. Second, also under field conditions a dissolved organic chemical would likely interact with suspended particles. That would influence the reaction mechanisms for either hydrolysis or photolysis. It is necessary to investigate the influence of dissolved organic matter for the degradation. Third, chemical units would have to be used for kinetics and mechanism to establish the chemical stoichiometry for kinetics. At last, about the toxicity and security of degradation product, it will be taken into consideration in the next step of work.

## 4. Conclusions

The hydrolysis and photolysis of FBEZF in water were studied in this paper. The effects of different factors were investigated in detail. The results showed that pH and temperature played critical roles in the degradation rate of FBEZF in water. The degradation rate of FBEZF in water decreased with pH, FBEZF rapidly degraded in alkaline aqueous solutions, and temperature substantially accelerated the degradation. Thermodynamic parameters were also obtained for hydrolysis of FBEZF under four hydrolysis conditions. The dissipation rate of FBEZF was hardly affected by the initial concentration. According to the result of the experiments, the degradation of FBEZF in water was relatively fast, and the half-lives of FBEZF were 14.44 d at pH = 5, and lower than 3 days under the other conditions studied. When FBEZF was applied to the field, it should be relatively safe due to its rapid degradation. The degradation rate of FBEZF in a water environment is relatively fast, so it won’t cause harm for the environment and human health. Moreover, the degradation product and mechanism of FBEZF were proposed. The degradation product was identified as 2-(4-fluorobenzyl)-5-methoxy-1,3,4-oxadiazole by NMR and MS. The degradation mechanism indicated that nucleophilic attack on the sulfone group and combination of the resulting intermediate combined with methanol, formed the degradation product by the loss of one H_2_O. The study of FBEZF’s degradation kinetics and degradation mechanism can contribute to its safety assessment and increase our understanding of the behavior of FBEZF in water environments.

## Figures and Tables

**Table 1:** Thermodynamic parameters for hydrolysis of FBEZF.

Kelvin Temperature (K)	288	298	308	318	Average
Rate constant k	0.009	0.018	0.035	0.231	/
Ea (kJ mol^−1^)	50.7	50.74	50.74	47.4	49.90
ΔH (kJ mol^−1^)	48.31	48.27	48.18	44.76	47.38
ΔS (kJ mol^−1^ *K)	−106.36	−114.8	−123.25	−131.69	−119.03

## Supplementary Materials

Supplementary Materials: The following are available online at http://www.mdpi.com/1660-4601/15/12/2741/s1, Figure S1. 1H NMR spectrum of degradation product P1; Figure S2. 13C NMR spectrum of degradation product P1; Figure S3. 19F NMR spectrum of degradation product P1; Figure S4. Mass Spectrum of degradation product P1; Table S1. Hydrolysis parameters of FBEZF in water with different pH values; Table S2. Hydrolysis parameters of FBEZF with different initial concentrations in water. Table S3. Hydrolysis parameters of FBEZF in water under different temperature; Table S4. Photolysis parameters of FBEZF with different initial concentrations in water.
