# Supplementary material for: Hydrolysis and Photolysis Kinetics, and Identification of Degradation Products of the Novel Bactericide 2-(4-Fluorobenzyl)-5-(Methylsulfonyl)-1,3,4-Oxadiazole in Water
Source: Int J Environ Res Public Health. 2018 Dec 5;15(12):2741. doi: 10.3390/ijerph15122741 (PMC6313691; doi:10.3390/ijerph15122741)
Supplement: Supplementary file 1 [file ijerph-15-02741-s001.pdf]

International Journal of
Environmental Research and Public Health

Supplementary Materials

Hydrolysis and Photolysis Kinetics, and Identification of Degradation Products of the Novel Bactericide 2-(4-Fluorobenzyl)-5-(Methylsulfonyl)-1,3,4-Oxadiazole in Water

Xingang Meng, Lingzhu Chen, Yuping Zhang, Deyu Hu * and Baoan Song *

^{1}H NMR, ^{13}C NMR, ^{19}F NMR spectra of FBEZF's degradation product P_1 (2-(4-fluorobenzyl)-5-methoxy-1,3,4-oxadiazole) were recorded on a JEOL ECX 500 NMR spectrometer operated at room temperature and 500 MHz using DMSO-d6 as a solvent and TMS as an internal standard. MS spectra were recorded on LC/MSD Trap VL.

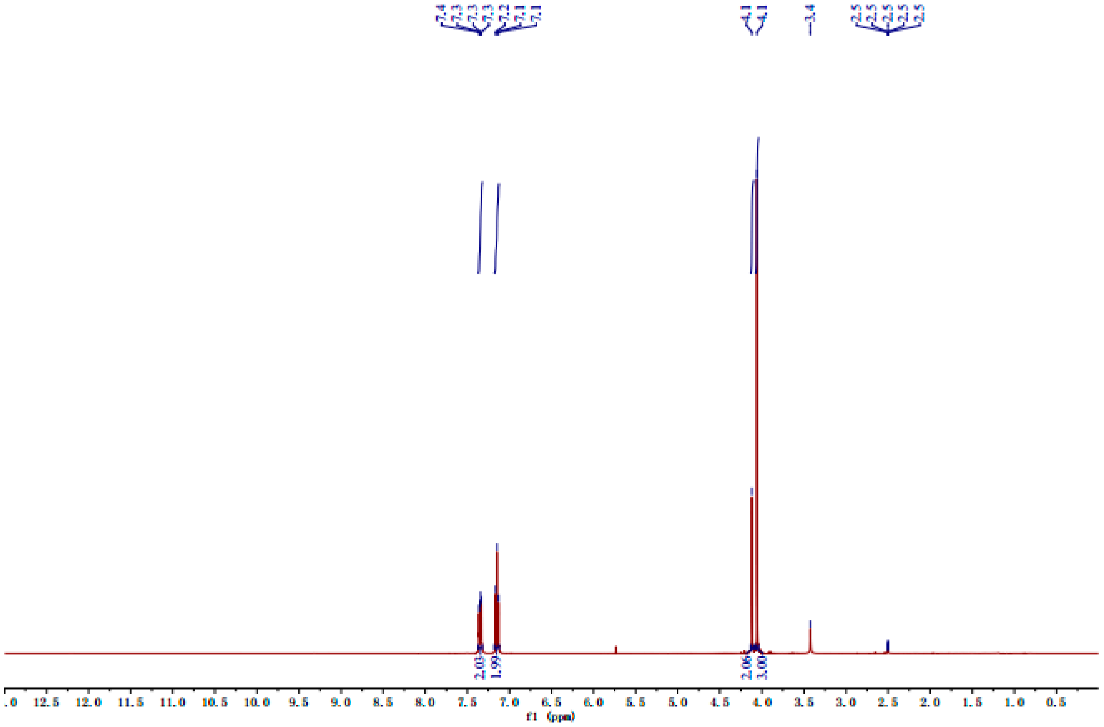

Figure S1. ^{1}H NMR spectrum of degradation product P_1.

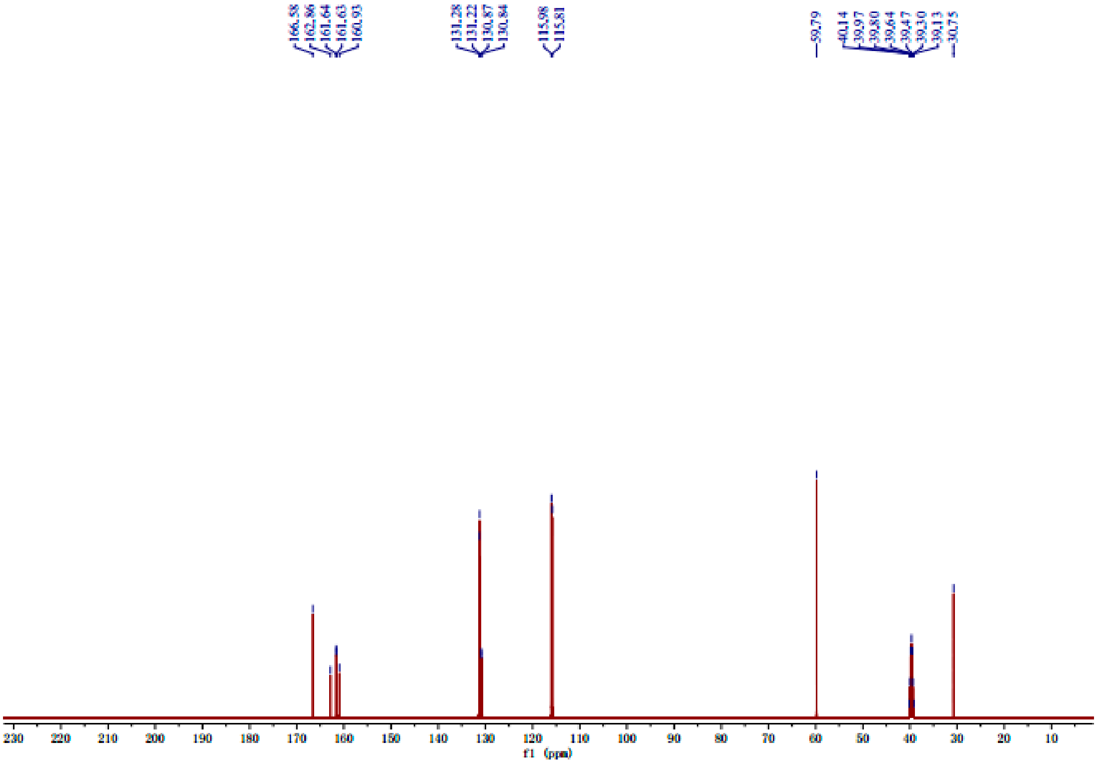

Figure S2. ^{13}C NMR spectrum of degradation product P_1.

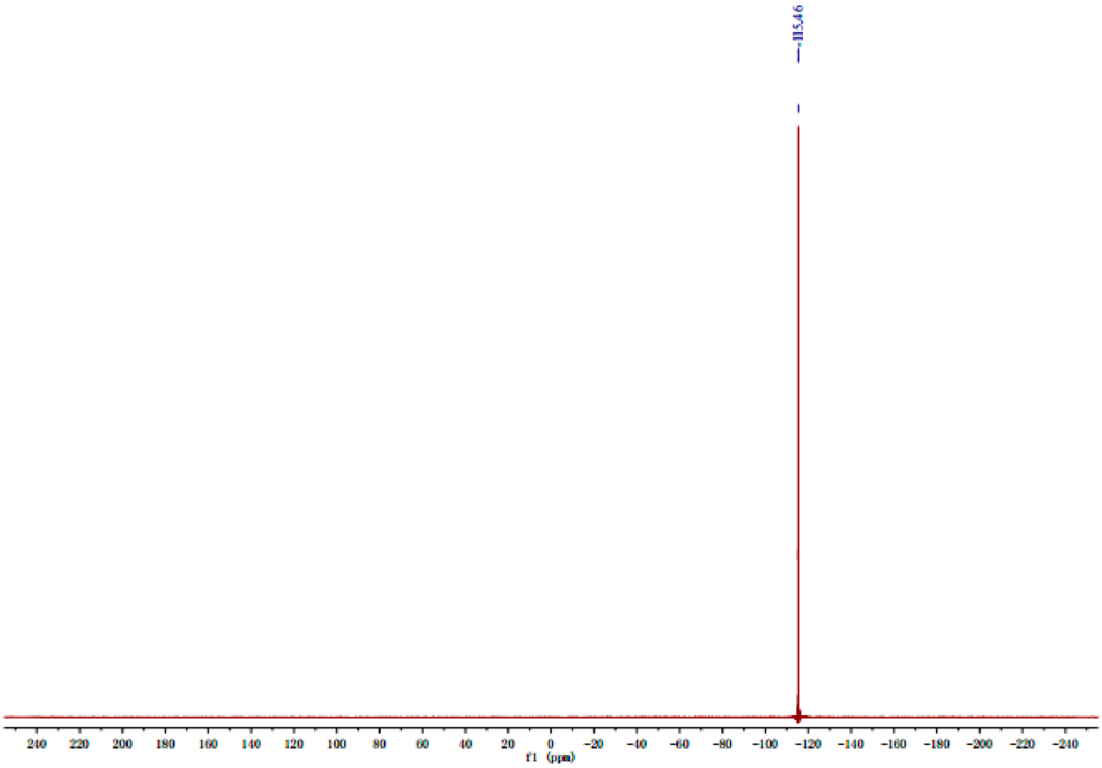

Figure S3. ^{19}F NMR spectrum of degradation product P_1.

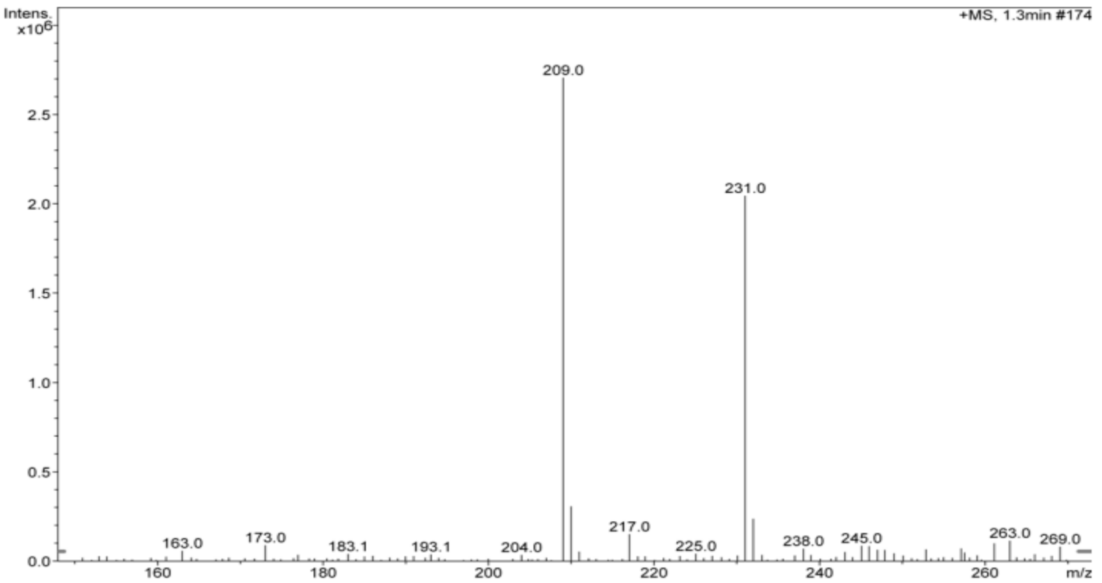

Figure S4. Mass Spectrum of degradation product P_1.

Table S1. Hydrolysis parameters of FBEZF in water with different pH values.

Initial concentration (mg L^{-1})	Temperature (°C)	pH	Kinetic equation	K (d^{-1})	Half-life (d)	R^2
5.0	25	5	$C_t = 5.0386e^{-0.048t}$	0.048	14.44	0.9832
5.0	25	7	$C_t = 4.9574e^{-0.432t}$	0.432	1.60	0.9972
5.0	25	9	/	/	/	/

Table S2. Hydrolysis parameters of FBEZF with different initial concentrations in water.

Initial concentration (mg L^{-1})	Temperature (°C)	pH	Kinetic equation	K (h^{-1})	Half-life (h)	R^2
1.0	25	7	$C_t = 0.7419e^{-0.019t}$	0.019	36.48	0.9869
5.0	25	7	$C_t = 4.9754e^{-0.018t}$	0.018	38.51	0.9972
10.0	25	7	$C_t = 11.765e^{-0.022t}$	0.022	31.51	0.9979

Table S3. Hydrolysis parameters of FBEZF in water under different temperature.

Initial concentration (mg L^{-1})	Temperature (°C)	pH	Kinetic equation	K (h^{-1})	Half-life (h)	R^2
5.0	15	7	$C_t = 4.9709e^{-0.009t}$	0.009	77.02	0.9949
5.0	25	7	$C_t = 4.9754e^{-0.018t}$	0.018	38.51	0.9972
5.0	35	7	$C_t = 4.8602e^{-0.035t}$	0.035	19.80	0.9967
5.0	45	7	$C_t = 5.5072e^{-0.231t}$	0.231	3.00	0.9948

Table S4. Photolysis parameters of FBEZF with different initial concentrations in water.

Initial concentration (mg L^{-1})	Temperature (°C)	pH	Kinetic equation	K (h^{-1})	Half-life (h)	R^2
1.0	25	7	$C_t = 0.9655e^{-0.079t}$	0.079	8.77	0.9892
5.0	25	7	$C_t = 4.2420e^{-0.083t}$	0.061	8.35	0.9909
10.0	25	7	$C_t = 9.7266e^{-0.080t}$	0.080	8.66	0.9955
